# Fibronectin extra domain A (FN-EDA) causes glaucomatous trabecular meshwork, retina, and optic nerve damage in mice

**DOI:** 10.1186/s13578-022-00800-y

**Published:** 2022-05-26

**Authors:** Timur A. Mavlyutov, Justin J. Myrah, Anil K. Chauhan, Yang Liu, Colleen M. McDowell

**Affiliations:** 1 Department of Ophthalmology and Visual Sciences, University of Wisconsin-Madison, Madison, WI USA; 2 Department of Internal Medicine, Division of Hematology/Oncology, University of Iowa, Iowa City, IA USA; 3 Department of Pharmacology and Neuroscience, North Texas Eye Research Institute, University of North Texas Health Science Center, Fort Worth, TX USA

**Keywords:** FN-EDA, Trabecular meshwork, Optic nerve, Glaucoma

## Abstract

**Background:**

Elevated intraocular pressure (IOP) is a major risk factor for the development and progression of primary open angle glaucoma and is due to trabecular meshwork (TM) damage. Here, we investigate the role of an endogenous Toll-like receptor 4 (TLR4) ligand, FN-EDA, in the development of glaucoma utilizing a transgenic mouse strain (B6.EDA^+/+^) that constitutively expresses only FN containing the EDA isoform.

**Methods:**

Eyes from C57BL6/J (wild-type), B6.EDA+/+ (constitutively active EDA), B6.EDA-/- (EDA null) mice were processed for electron microscopy and consecutive images of the entire length of the TM and Schlemm’s canal (SC) from anterior to posterior were collected and montaged into a single image. ECM accumulation, basement membrane length, and size and number of giant vacuoles were quantified by ImageJ analysis. Tlr4 and Iba1 expression in the TM and ONH cells was conducted using RNAscope in situ hybridization and immunohistochemistry protocols. IOP was measured using a rebound tonometer, ON damage assessed by PPD stain, and RGC loss quantified in RBPMS labeled retina flat mounts.

**Results:**

Ultrastructure analyses show the TM of B6.EDA^+/+^ mice have significantly increased accumulation of ECM between TM beams with few empty spaces compared to C57BL/6 J mice (p < 0.05). SC basement membrane is thicker and more continuous in B6.EDA^+/+^ mice compared to C57BL/6 J. No significant structural differences are detected in the TM of EDA null mice. *Tlr4* and Iba1 expression is increased in the TM of B6.EDA^+/+^ mice compared to C57BL/6 J eyes (p < 0.05). IOP is significantly higher in B6.EDA^+/+^ mice compared to C57BL/6 J eyes (p < 0.001), and significant ON damage (p < 0.001) and RGC loss (p < 0.05) detected at 1 year of age. *Tlr4* mRNA is expressed in mouse ONH cells, and is present in ganglion cell axons, microglia, and astrocytes. There is a significant increase in the area occupied by Iba-1 positive microglia cells in the ONH of B6.EDA^+/+^ mice compared to C57BL/6 J control eyes (p < 0.01).

**Conclusions:**

B6.EDA^+/+^ mice have increased ECM accumulation in the TM, elevated IOP, enhanced proinflammatory changes in the ONH, loss of RGCs, and ONH damage. These data suggest B6.EDA^+/+^ mice recapitulate many aspects of glaucomatous damage.

**Supplementary Information:**

The online version contains supplementary material available at 10.1186/s13578-022-00800-y.

## Background

It is well known that elevated intraocular pressure (IOP) is a primary risk factor for the development as well as the progression of glaucoma. Glaucoma is characterized by cupping of the optic nerve head (ONH), thinning and loss of the retinal nerve fiber layer, and characteristic visual field defects. Primary open angle glaucoma (POAG) is the most common form of glaucoma and is characterized by elevated IOP due to increased aqueous humor outflow resistance. The majority of aqueous humor drains from the eye through the conventional outflow pathway, passing through the trabecular meshwork (TM), Schlemm’s canal (SC), and eventually into the episcleral veins. The TM and SC regions are the primary determinant and homeostatic regulator of IOP [1, 2]. The TM comprises semi-fenestrated beams of connective lamina with incorporated flat cells of neural crest origin and epithelial morphology [1]. The outer part of the TM forms the juxtacanalicular tissue (JCT) with abundant ECM, which is located adjacent to the inner wall of SC. Increased deposition of ECM proteins in the TM, as well as the stiffness of the ECM and TM cells, is believed to be the cause of increased AH outflow resistance and increased IOP associated with POAG [3, 4]. In addition, changes to the ECM underlying the inner wall of Schlemm’s canal (SC) as well as an increased stiffness has also been identified as a major contributing cause to elevated IOP [2]. In order to maintain ECM integrity, TM cells continually secrete a wide variety of ECM and metalloproteases [5]. Profibrotic TGFβ2 signaling leads to an increase in the deposition of fibronectin [6], fine fibrillar material [7, 8] and altered glycosaminoglycan composition [9] leading to damage to the ECM in the TM and an increased stiffness [10, 11]. These data establish that the ECM makeup of the TM is important in controlling aqueous humor outflow and IOP.

In particular, the multidomain ECM glycoprotein fibronectin (FN) has previously been shown to be elevated in glaucomatous TM tissues and AH [12–15]. FN has multiple functions both in regulating cellular processes as well as guiding and maintaining tissue organization and ECM composition. FN plays an important role in directing ECM-ECM and ECM-cell interactions, as well as regulating ECM remodeling through activation of associated growth factors and proteins. FN is composed of type I, type II, and type III domains and has over 20 alternatively spliced isoforms. FN is composed of either cellular FN (cFN) or plasma FN (pFN). Here we focus on cFN and the isoform extra domain A (EDA) [16]. It is known that the fibronectin EDA (FN-EDA) isoform is abundant throughout embryonic development [17]; however, in adults FN-EDA expression is typically minimal. The primarily function of FN-EDA in adult tissue is as a structural scaffold and ECM-cell signaling molecule that regulates cell migration, adhesion, and proliferation [18]. Importantly, it is well known that the expression of FN-EDA is upregulated in disease states and as a response to tissue repair, injury, or remodeling [19–22].

In addition, it is known that both FN as well as the FN-EDA isoform are significantly elevated in human glaucomatous TM [23]. We have previously shown that TGFβ2-induced ocular hypertension is dependent on both FN-EDA and its receptor toll-like receptor 4 (TLR4) [13, 24, 25]. Functionally, it is known that EDA acts as an endogenous ligand (DAMP, damage associated molecular pattern) for TLR4, eliciting similar downstream responses as LPS [26]. DAMP activated TLR4 signaling has been linked to fibrosis and the regulation and production of ECM proteins in hepatic fibrosis, renal fibrosis, lesional skin and lung in scleroderma patients, and most important to the work presented here we have linked DAMP activated TLR4 to TM damage and ocular hypertension [13, 27–30]. TLR4 activation downregulates the TGFβ antagonist, BMP and the activin membrane-bound inhibitor (BAMBI), which enhances TGFβ signaling leading to increased ECM production [27, 28] and production of DAMPs such as FN-EDA [24].

Previously, we demonstrated in primary human TM cells in culture, that inhibition of TLR4 blocks TGFβ2-induced ECM production, and FN-EDA enhanced TGFβ2-induced ECM production [13]. In addition, we showed that mice constitutively expressing the EDA isoform develop ocular hypertension [24]. There are several mouse models of ocular hypertension and glaucoma described in the literature and each has its strengths and weaknesses as previously described and reviewed [31–39]. The most relevant models recapitulate the human condition with similar phenotypes within the conventional outflow pathway including damage to the TM and SC which generates elevated IOP and outflow dysfunction leading to RGC and ON damage [40]. However, many of the current models either rely on occluding the aqueous outflow with substances such as microbeads or silicon/HA substrates, involve injection of adenoviruses to overexpress glaucoma-relevant genes, represent acute models’ systems rather than disease-relevant chronic development of disease pathology, or have complex mixed genetic backgrounds which make interpretation of the data more difficult and involved time-consuming breeding paradigms [31, 40, 41]. Here we identify a human glaucoma relevant mouse model in which mice constitutively expressing FN-EDA display disease relevant ocular hypertension and glaucoma phenotypes including accumulation of ECM in the TM, changes to SC basement membrane and giant vacuoles, elevated IOP, increased ONH microglial activation, ON damage, and loss of RGCs. These data identify a novel mouse model system to study the molecular pathology of the TM as well as glaucomatous damage to the retina, optic nerve head, and optic nerve.

## Results

**Fig. 1:**
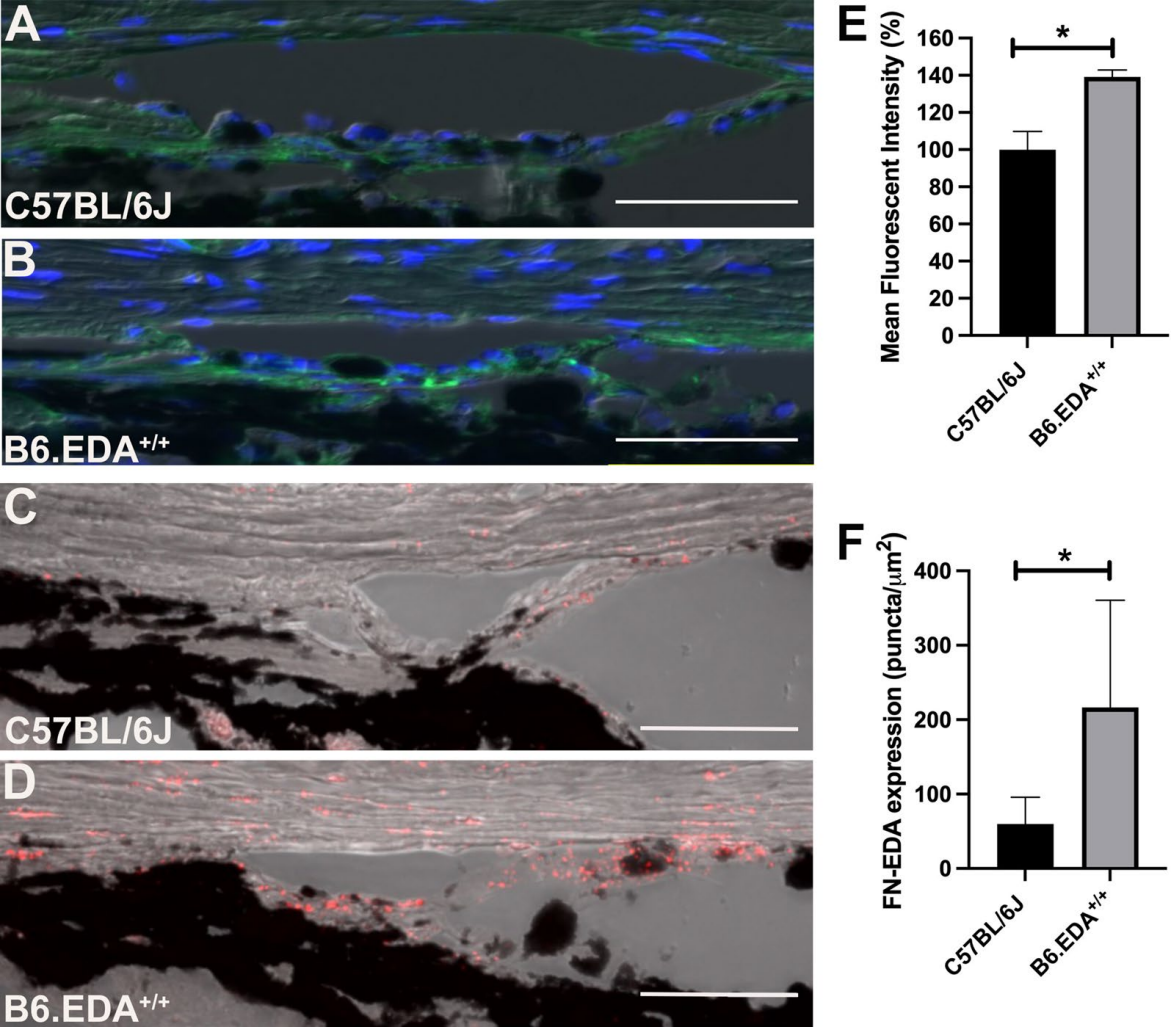
Total Fibronectin and Fibronectin-EDA expression is increased in the TM of B6.EDA^+/+^ mice. **A**, **B** Total FN expression is increased in the TM of B6.EDA^+/+^ mice compared to C57BL/6J controls as shown by immunohistochemical analysis (green labeling). **C**, **D** The EDA isoform of FN is increased in the TM of B6.EDA^+/+^ mice compared to C57BL/6J controls as shown by RNAscope in situ hybridization (red puncta). Both total FN (**E**) and FN-EDA (**F**) were significantly increased when quantified by ImageJ analysis of mean fluorescent intensity and puncta/mm^2^, respectively. Significance determined by Student’s t-test. *p < 0.05, n = 3 mice/strain, scale bar = 50 µm

Previously, we reported that B6.EDA^+/+^ mice have elevated IOP and increased total FN and FN containing the EDA isoform compared to C57BL/6J controls [24]. In addition, we showed normal open angle development in B6.EDA^+/+^ mice by gross clinical and histological exams at 15, 30, and 60 days post-natal. Here, we recapitulate this data at 7 months of age in B6.EDA^+/+^ and C57BL/6J mice with immunostaining of total FN (Fig. 1A, B) and in situ hybridization of the EDA isoform of FN (Fig. 1C, D). Both FN and the EDA isoform were significantly increased in the TM of B6.EDA^+/+^ mice compared to controls (Fig. 1E, F).

**Fig. 2:**
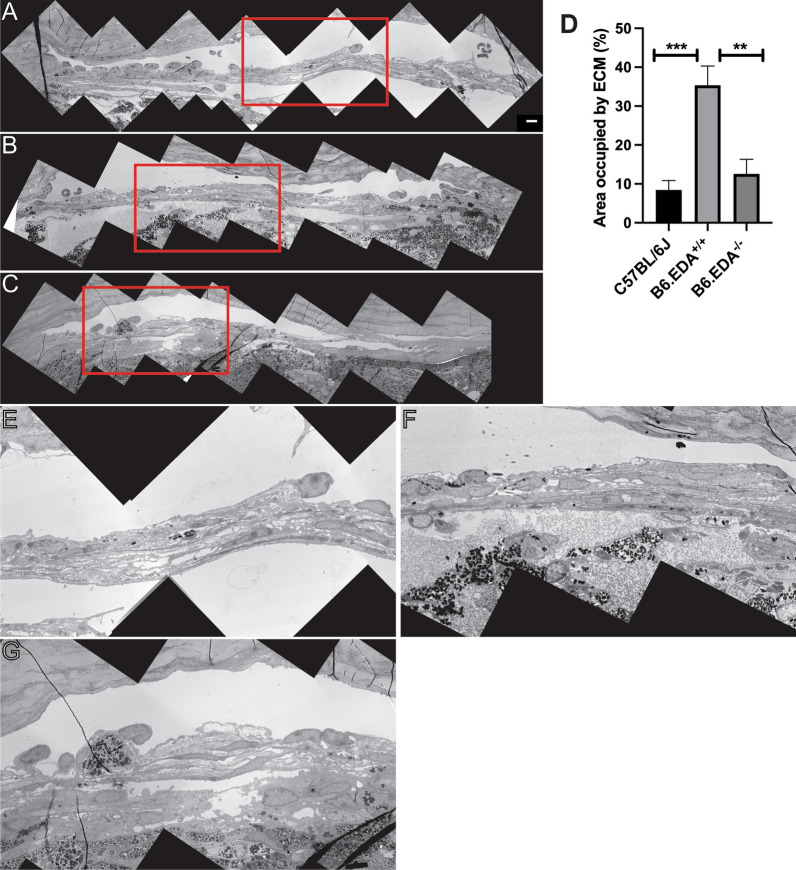
ECM accumulation is increased in the TM of B6.EDA^+/+^ mice. **A**, **B**, **C** Montage of low magnification images (2650×) shows entire TM and inner part of SC in eyes of C57BL/6 J control (**A**), B6.EDA^+/+^ (**B**), and EDA^−/−^ mice (**C**). Regions outlined in the red box in upper panels are enlarged in lower panels (C57BL/6J (**E**), B6.EDA^+/+^ (**F**), and B6.EDA^−/−^ (**G**)). Throughout the entire length of the TM fewer empty spaces (green asterisks) exist in the TM of B6.EDA^+/+^ eyes compared to C57BL/6J controls. A network of ECM surrounds the inner most part of the TM in B6.EDA^+/+^ eyes (red arrows), while it is almost absent in C57BL/6J control eyes. **D**. Area occupied by ECM in the TM was significantly increased in B6.EDA^+/+^ eyes compared to C57Bl/6J and B6.EDA^−/−^ eyes. Significance determined by one-way ANOVA, *** = p < 0.001, ** = p < 0.01, n = 3 mice/strain, two planes of stitched sections along the entire length of SC analyzed per mouse, scale bar = 5 µm

To further analyze the changes to the TM and ECM at the ultrastructural level, transmission electron microscopy studies were employed. The TM of B6.EDA^+/+^ mice have fewer empty spaces between the trabecular beams, increased deposition of ECM throughout the TM, and the inner side of the TM has increased accumulation of ECM (Fig. 2B, F) compared to the C57BL/6J control mice (Fig. 2A, E ). The ultrastructure of the TM in FN-EDA null mice (B6.EDA^−/−^) was comparable to that of the normal C57BL/6J mice with similar pattern of open spaces and little ECM deposition (Fig. 2C, G). B6.EDA^+/+^ mice have a significantly increased amount of ECM in the TM compared to C57BL/6J mice and B6.EDA^−/−^ as quantified by ImageJ analysis, significance determined by one-way ANOVA (Fig. 2D).

**Fig. 3:**
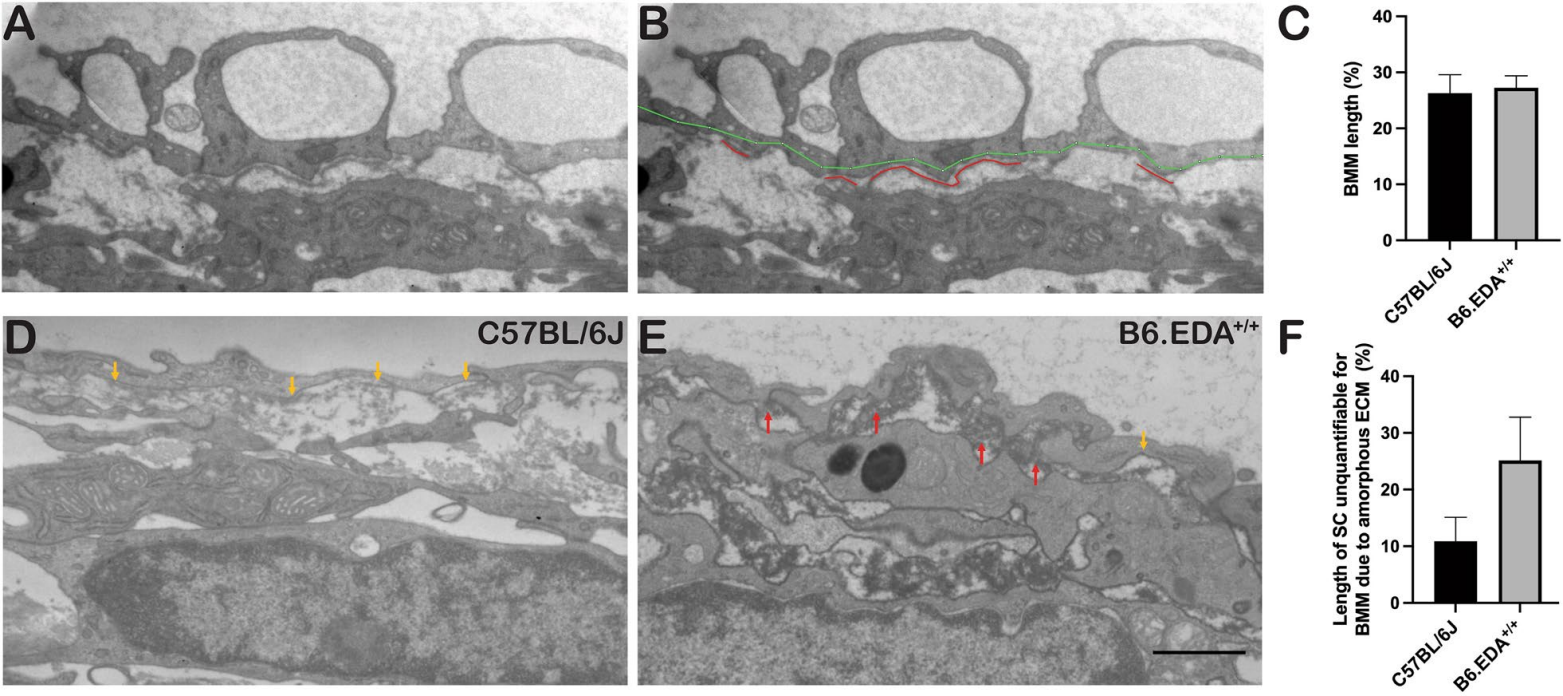
B6.EDA^+/+^ mice have increased amorphous ECM and changes to basement membrane material along the inner wall of SC. **A**, **B** Fragment of the stitched image of the entire length of SC (**A**) and a representative example of how the percent length of BMM along the inner wall of SC was measured (**B**). The inner wall of SC is marked with a green line and BMM marked with red a line. **C** Percent of BMM covering the length of the SC in C57BL/6J and B6.EDA^+/+^ mice. **D**, **E** Large portions of the inner SC endothelium is covered by amorphous ECM material (red arrows) in B6.EDA^+/+^ mice, which may conceal the BMM (yellow arrows), and makes it more difficult to detect of BMM in these regions. **F** Length of SC that is unquantifiable for BMM due to amorphous material under the endothelium in C57BL/6J and B6.EDA^+/+^ mice. n = 3 mice/strain, two planes of stitched sections along the entire length of SC analyzed per mouse, scale bar = 1 µm.

In the normal eye, most of the outflow resistance is suggested to occur at the inner wall endothelium of Schlemm’s canal (SC), its discontinuous basement membrane, and the juxtacanalicular connective tissue (JCT). The density and amount of basement membrane materials (BMM) underlying the inner wall of SC has been shown to be associated with elevated IOP and increased outflow resistance [1, 42, 43]. Here we analyzed the amount of BMM in B6.EDA^+/+^ mice and C57BL/6J controls (Fig. 3). Qualitatively we observed a thicker amount of amorphous material under the endothelial cells along the inner wall of SC in B6.EDA^+/+^ mice (Fig. 3E) in comparison to C57BL/6J control mice (Fig. 3D). We quantified the amount of BMM below the inner wall of SC following the approach described by Overby et al. [42] (Fig. 3A, B). This method measures the fraction of inner wall length exhibiting continuous basement membrane material, identified as a thin line of basal lamina-like structure underlying the inner wall endothelium of SC [42]. The length of the basement membrane material was measured in stitched contiguous sagittal sections, imaged at 12000×, spanning the entire anterior-to-posterior length of SC. Interestingly, we did not find a significant difference in BMM material (Fig. 3C). The significant amount of amorphous-like ECM material throughout the TM and JCT region in B6.EDA^+/+^ mice made it very difficult to quantify the BMM utilizing the method described by Overby et al. [42], and it is very likely we are underestimating the amount of BMM. Indeed, over 25% of the length of SC was unquantifiable for BMM due to the amorphous ECM impeding the analysis region (Fig. 3F).

**Fig. 4:**
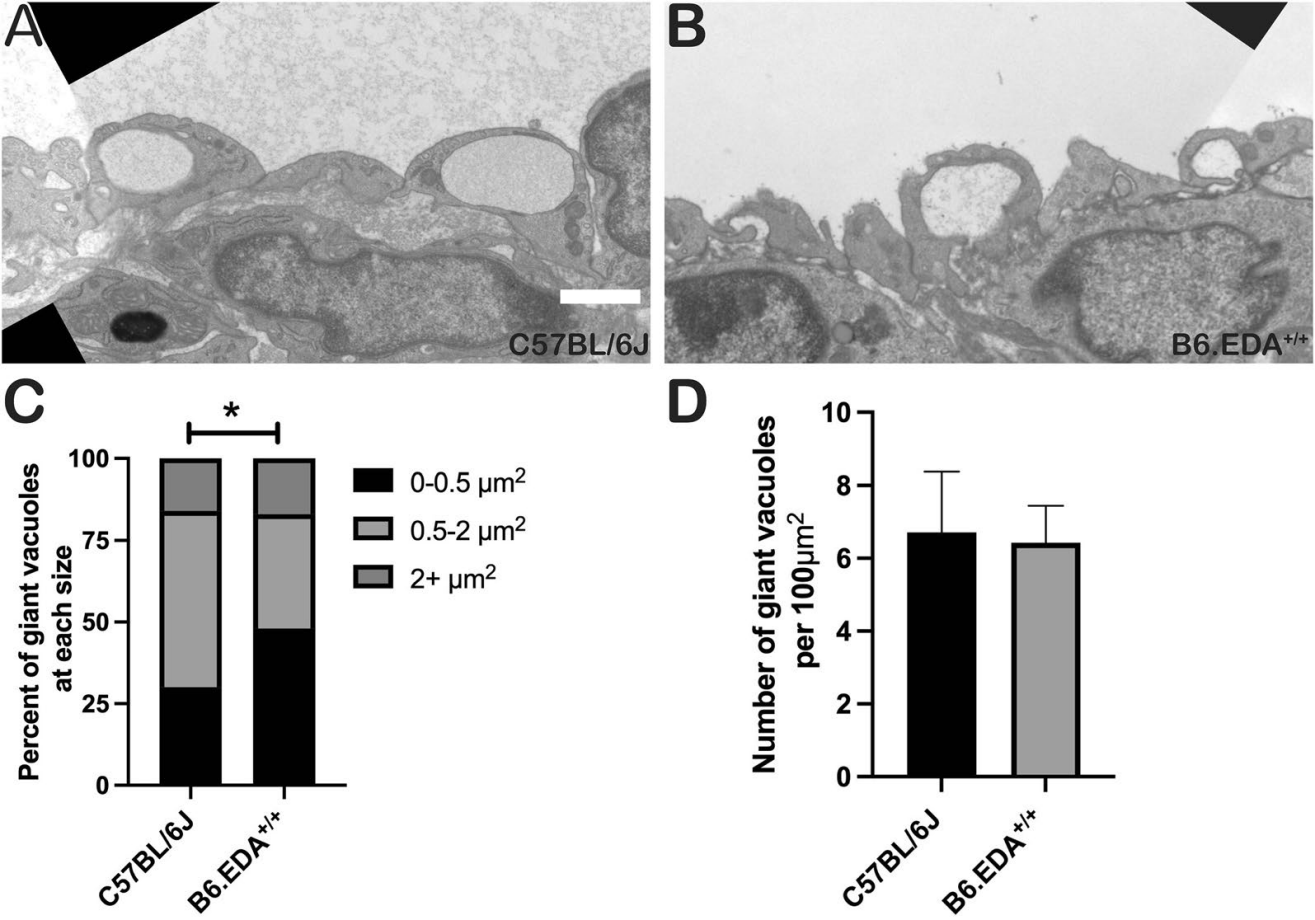
B6.EDA^+/+^ mice have smaller giant vacuoles. **A**, **B** Fragment of the stitched image of the entire length of SC of C57BL/6J (**A**) and B6.EDA^+/+^ (**B**) mice. **C** Quantified percent of giant vacuoles with various sizes from C57BL/6J and B6.EDA^+/+^ mice. B6.EDA^+/+^ mice had significantly more smaller giant vacuoles. **D** Total number of giant vacuoles per 100 μm^2^. Significance determined by Chi-square, p < 0.05, n = 3 mice/strain, two planes of stitched sections along the entire length of SC analyzed per mouse, scale bar = 2 µm

Giant vacuoles are involved in the transport of aqueous humor across the inner wall of SC. Here, we analyzed the size and number of giant vacuoles across the entire anterior to posterior length of SC, measured at 12000× (Fig. 4A, B). B6.EDA^+/+^ mice had significantly more small giant vacuoles compared to C57BL/6J controls (Fig. 4C), but similar overall numbers of giant vacuoles between the mouse strains (Fig. 4D).

**Fig. 5:**
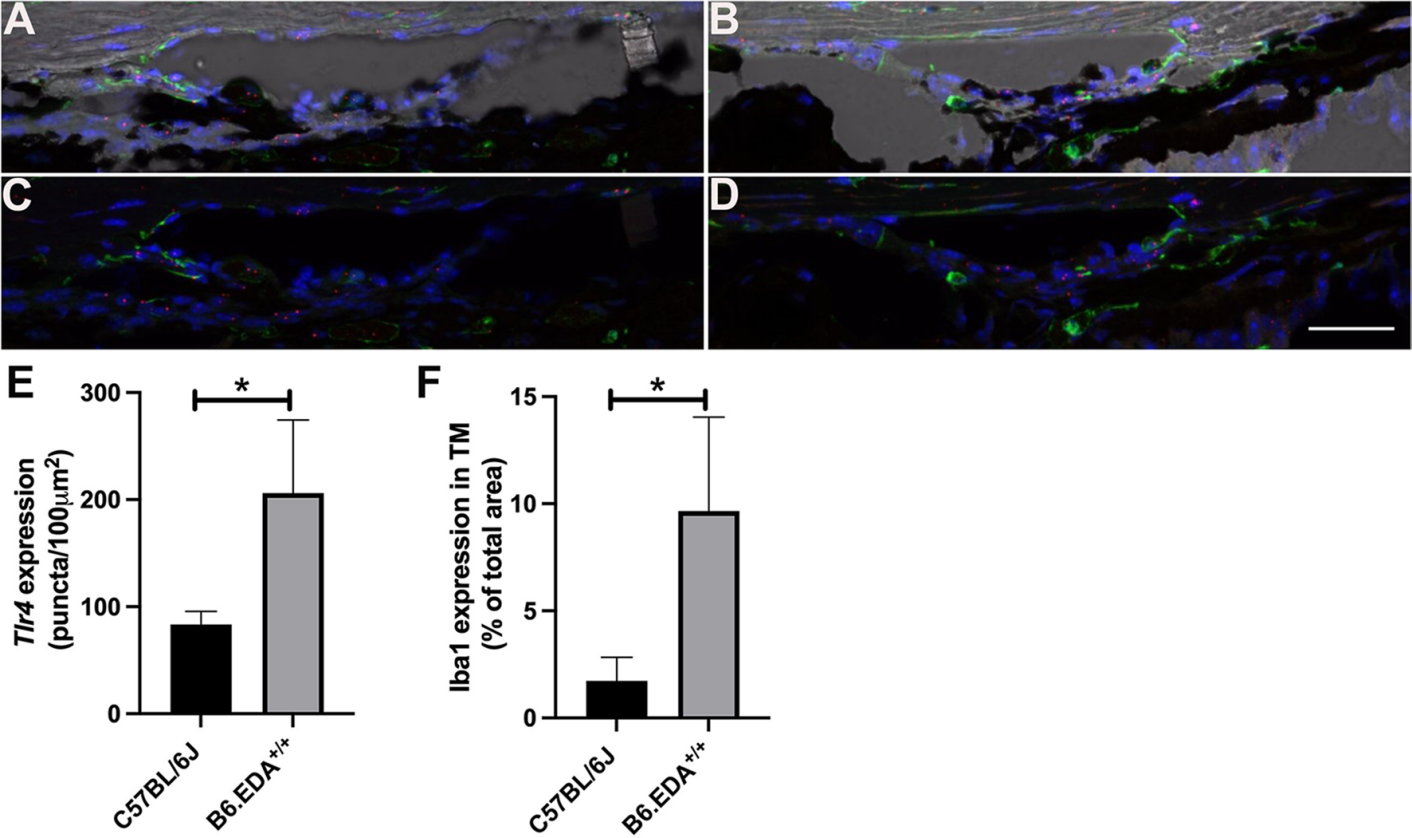
B6.EDA^+/+^ mice have increased expression of *Tlr4* and Iba1 in the TM. **A**, **B** Merged fluorescent and brightfield images show increased expression of *Tlr4* mRNA by in situ hybridization and Iba1 by immunofluorescent labeling in the TM of B6.EDA^+/+^ mice (**B**) compared to controls (**A**). Unmerged images shown for C57BL/6J (**C**) and B6.EDA^+/+^ (**D**). Puncta of *Tlr4* mRNA is red, Iba-1 positive macrophages are green, blue is DAPI nuclear stain. Scale = 100 µm. **E** Total *Tlr4* mRNA puncta in TM of C57BL/6J and B6.EDA^+/+^ mice quantified by ImageJ analysis. **D** Quantified coverage of TM area by Iba-1 positive expression in C57BL/6J and B6.EDA^+/+^ mice. Significance determined by Student’s t-test, *p < 0.05, n = 3 mice/strain

Previously, we identified that EDA-induced ocular hypertension was dependent on TLR4 [24]. Mice harboring both the constitutively active EDA isoform and knock out alleles of TLR4 (B6.EDA^+/+^TLR4^−/−^ mice) exhibited normal IOP and were resistant to TGFβ2-induced ocular hypertension. These data suggest that EDA is acting as a DAMP for TLR4 activation. Here, we evaluated the levels of *Tlr4* mRNA expression in the TM of C57BL/6J control and B6.EDA^+/+^ mice (Fig. 5). *Tlr4* mRNA (red puncta) is detected in the TM of both C57BL/6J control (Fig. 5A, C) and B6.EDA^+/+^ mice (Fig. 5B, D), and levels of *Tlr4* mRNA are significantly increased in the TM of B6.EDA^+/+^ mice (Fig. 5E). In addition, there are increased numbers of Iba1 positive macrophages in the TM of B6.EDA^+/+^ mice compared to control eyes (Fig. 5F), which may be relevant to a fibro-inflammatory response in the TM.

**Fig. 6:**
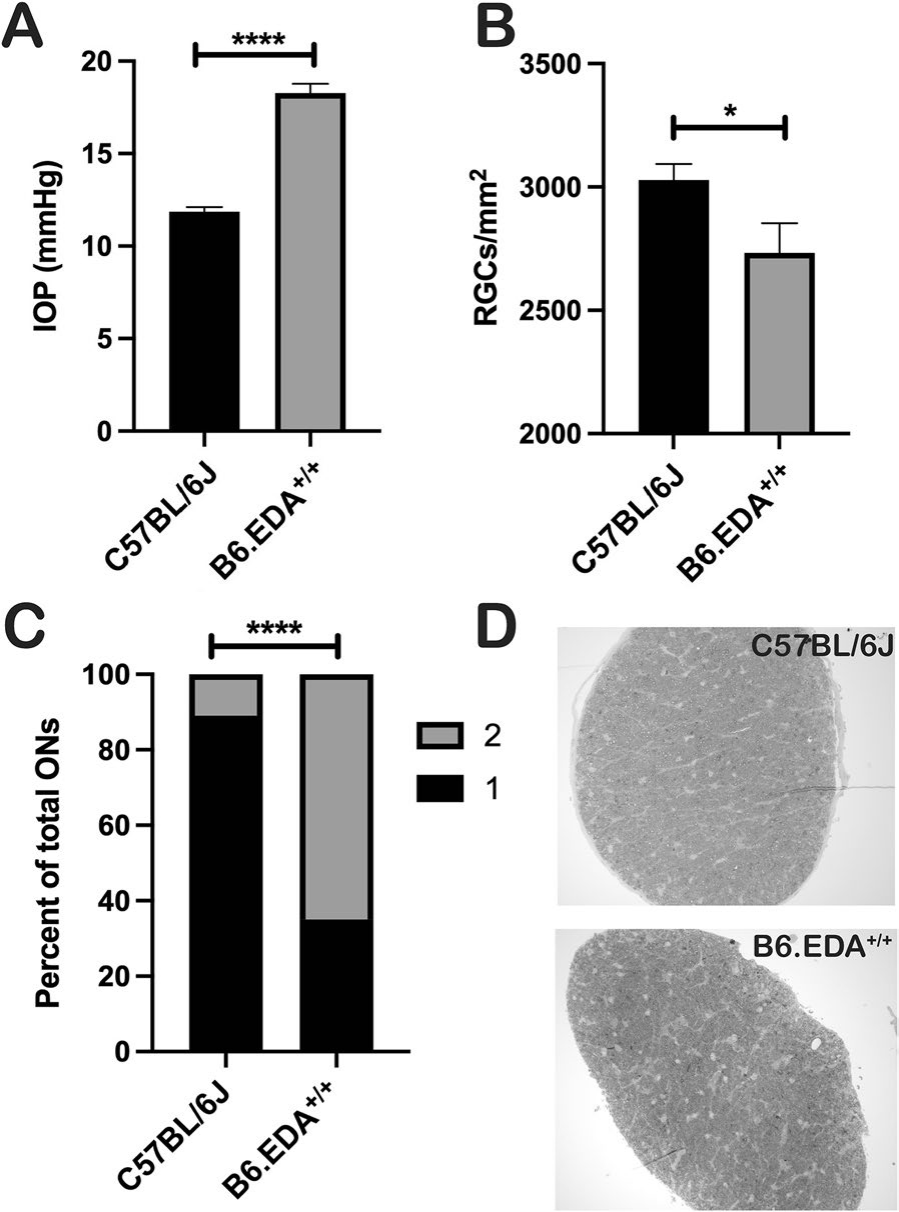
B6.EDA^+/+^ mice have elevated IOP, RGC loss, and ON damage. **A** IOP is significantly elevated in B6.EDA^+/+^ mice (n = 16) compared to C57BL/6J (n = 22) at 12 months of age, mean ± SEM. Significance determined by Student’s t-test. **B** B6.EDA^+/+^ mice (n = 10) have 9.7% loss of RGCs at 12 months of age compared to C57BL/6J (n = 15) as quantified by RBPMS stained retinal flat mounts. Significance determined by Student’s t-test (**C**) Percent of axons with no damage [1] or with mild damage (2) in transverse sections of optic nerves in B6.EDA^+/+^ (n = 23) and C57BL/6J (n = 18) mice. B6.EDA^+/+^ have significantly more ON damage compared to C57BL/6J, Significance determined by Fisher's exact test. **D** Images of representative transverse sections of optic nerves of B6.EDA^+/+^ and C57BL/6J mice. * = p < 0.05, **** = p < 0.0001

As previously reported, B6.EDA^+/+^ mice have significantly higher IOP pressure than C57BL/6J control mice [24]. Here, we recapitulate this data and demonstrate that the IOP remains significantly elevated in B6.EDA^+/+^ mice at 12 months of age (Fig. 6A). Since increased IOP is associated with the risk of damage to the RGC’s and axons, we analyzed RGC cell numbers in RBPMS labeled retina flat mounts and found a 9.8% decrease in RGC numbers in the B6.EDA^+/+^ retinas compared to C57BL/ 6J controls at 12 months of age (Fig. 6B). In addition, utilizing a well-established Optic Nerve Damage Scoring rubric [41], we quantified axonal integrity in the ON. ONs from B6.EDA^+/+^ and C57BL/6J control mice at 12 months of age were scored in a masked manner by two independent individuals. There is significantly more damage in the ONs of B6.EDA^+/+^ mice, with many ONs scoring a “2” indicative of mild ON damage and approximately 10% of darkly stained axons and initial stages of gliosis [41] (Fig. 6C, D).

**Fig. 7:**
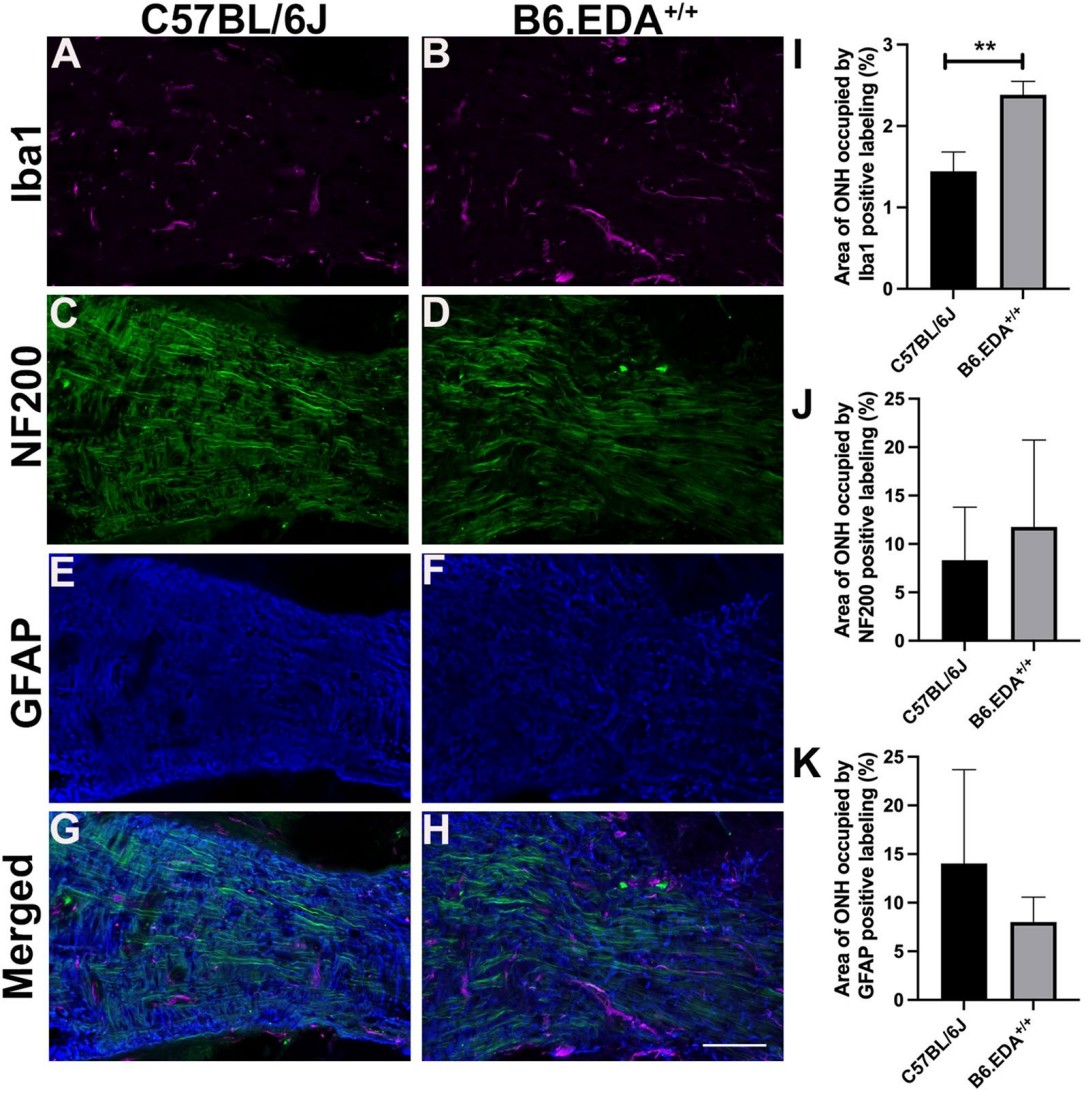
B6.EDA^+/+^ mice have increased Iba1 expression in the ONH. (A-H) immunohistochemistry staining for microglia (purple) by Iba1 antibody labeling (**A**, **B**), RGC axons (green) by NF200 antibody labeling (**C**, **D**), and astrocytes (blue) by GFAP antibody labeling (**E**, **F**) in the ONH of C57BL/6J and B6.EDA^+/+^ mice at 8–9 months of age. **A**, **B**, **I** B6.EDA^+/+^ mice have increased area covered by Iba-1 positive expression in the ONH compared to C57BL/6J controls. **C**, **D**, **J** No significant difference detected in coverage by NF200 labeled ganglion cell axons. **E**, **F**, **K** No significant difference detected in coverage by GFAP labeled astrocytes. Significance determined by Student’s t-test. Scale bar = 50 μm. n = 3 mice/strain

The ONH region also contains microglia and astrocytes, both of which have been shown to be important in the initial stages of glaucomatous damage. Here, we analyzed the expression of astrocyte marker GFAP, microglia marker Iba1, and axon marker NF200 in the ONH of C57BL/6J control and B6.EDA^ + / +^ mice at 8–9 months of age, (Fig. 7). There was a significant increase in the area occupied by Iba-1 positive microglia in the ONH of B6.EDA^+/+^ mice compared to controls (Fig. 7A, B, I). Increased Iba-1 expression is an indicator of increased microglia activation and density and is a known marker of early glaucomatous changes, proceeding RGC loss and ON damage [44–48]. GFAP was expressed throughout the ONH in C57BL/6J control and B6.EDA^+/+^ mice with no significant difference in the area occupied by GFAP (Fig. 7E, F, J). There was also no significant difference in NF200 labeled RGC axons as quantified by immunolabeling in cryopreserved cross-sections (Fig. 7C, D, K).

**Fig. 8:**
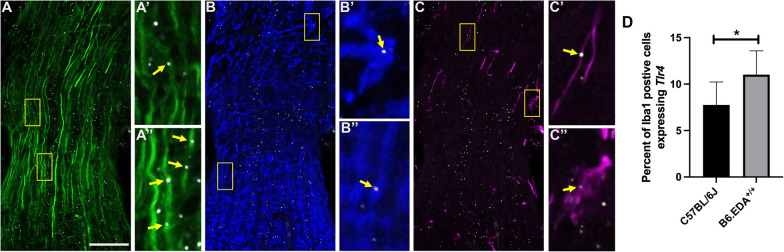
*Tlr4* is expressed in RGC axons, astrocytes, and microglia in the mouse ONH**.** *Tlr4* mRNA shown as white puncta in all images by RNAScope in situ hybridization in combination with immunofluorescent labeling of axons, astrocytes, and microglia. **A** *Tlr4* mRNA is detected in RGC axons (green), **B** in ONH astrocytes (blue) and **C** in ONH microglia (magenta). **A’** and **A”** are magnified fragments of **A**; **B’** and **B”** are magnified fragments of **B**; **C’** and **C”** are magnified fragments of **C**. Yellow arrows point to localization of *Tlr4* mRNA in cells positive for specific markers. **D** Bar graph showing percent of Iba1 positive cells with detected *Tlr4* mRNA in C57BL/6J control and B6.EDA^+/+^ mice. Significance determined by Student’s t-test. Scale = 50 µm. n = 3 mice/strain

**Fig. 9:**
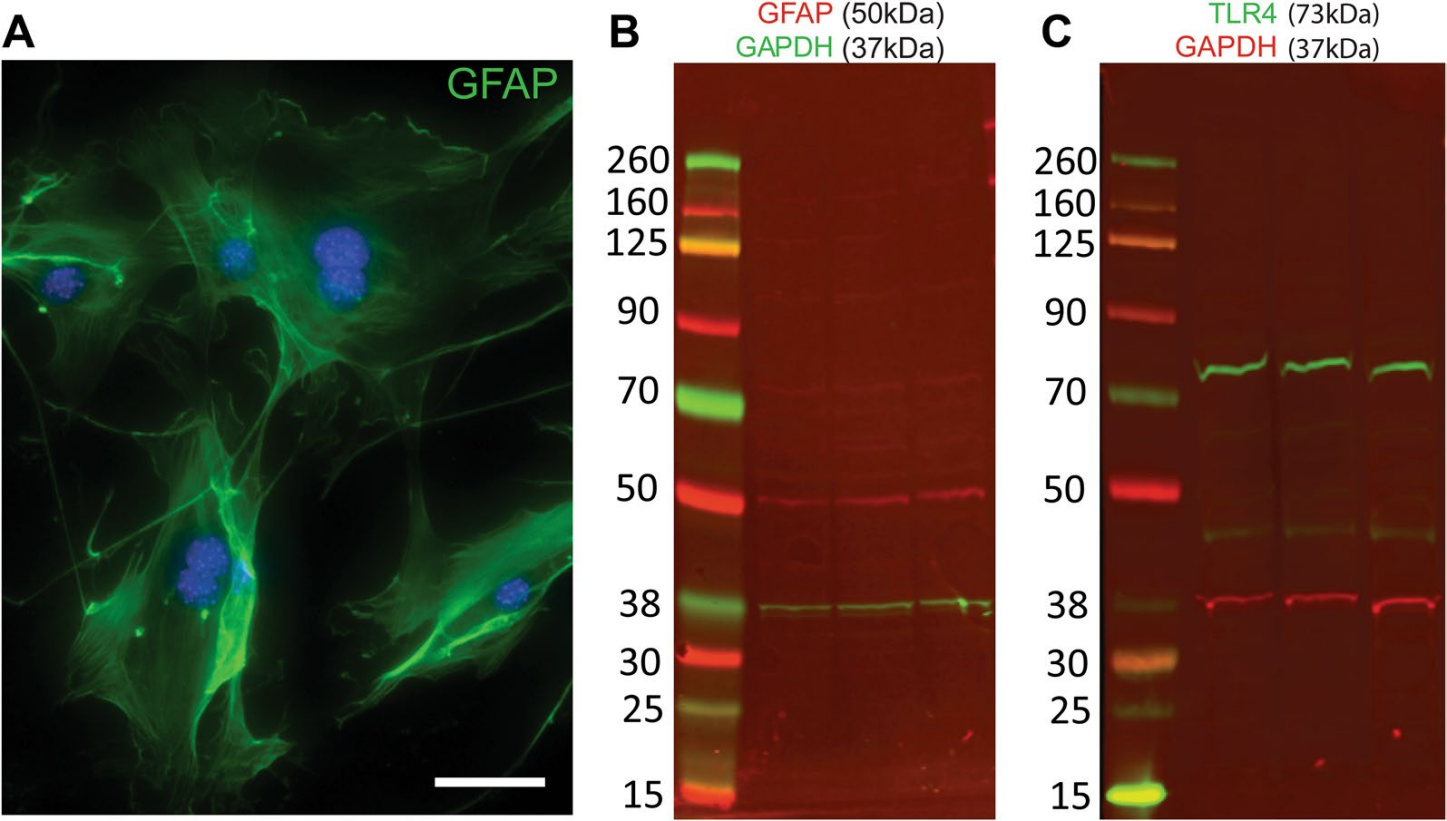
TLR4 is expressed in primary mouse ONH astrocytes. **A** Astrocytes isolated from the mouse ONH express astrocyte-specific marker GFAP. Scale bar = 50 μm. **B** Cell lysates were isolated from the primary astrocyte cultures and GFAP (50kD, red) and GAPDH (37kD, green) expression detected by western blot. **C** Cell lysates isolated from primary astrocyte cultures were isolated and TLR4 (73 kDa, green) and GAPDH (37kD, red) expression detected by western blot

Given the importance of FN-EDA in activation of TLR4, we determined the expression of *Tlr4* in the ONH cells of C57BL/6J and B6.EDA^+/+^ mice by RNAscope in situ hybridization. *Tlr4* mRNA is expressed in mouse RGC axons, microglia, and astrocytes (Fig. 8). The specificity of the *Tlr4* probe was validated using TLR4^−/−^ mice (Additional file 1: Figure SA1). No significant difference in *Tlr4* mRNA expression was detected in ONH astrocytes or RGC axons between C57BL/6J and B6.EDA^+/+^ mice. However, the percent of Iba1 positive cells expressing *Tlr4* mRNA was significantly increased in the ONH of B6.EDA^+/+^ mice compared to controls (Fig. 8D). We further evaluated the expression of TLR4 protein in primary astrocyte cultures derived from the mouse ONH tissue (Fig. 9). Astrocytes were characterized by detection of astrocyte-specific marker GFAP (Fig. 9A, B). Similar to the in-situ hybridization data (Fig. 8B) TLR4 protein was also detected in the isolated primary ONH astrocytes in culture (Fig. 9C).

## Discussion

Mice are genetically similar to humans and have a comparable ocular anatomy and physiology, including of the conventional outflow pathway, making them an ideal model system to study ocular hypertension and glaucoma [31, 49–55]. Importantly, mice can easily be genetically manipulated, specific genes can be targeted, and mouse genetics can be utilized to uncover modifier genes leading to various disease relevant phenotypes. Previously, we utilized several transgenic mouse strains to determine that both TLR4 and the EDA isoform of FN are necessary for TGFβ2-induced ocular hypertension [24]. In addition, we showed that constitutively active EDA mice (B6.EDA^+/+^) developed ocular hypertension by 14 weeks of age [24]. These data suggest that B6.EDA^+/+^ mice harbor important phenotypes of ocular hypertension and glaucomatous TM damage. Here, we further investigate the role of FN-EDA in generating elevated IOP by examining the ECM makeup and ultrastructure of the TM by light and electron microscopy. In addition, we demonstrate subsequent RGC loss and ON damage B6.EDA^+/+^ following sustained IOP elevation.

The ultrastructure changes in the TM of B6.EDA^+/+^ mice resemble the changes previously reported in other models of ocular hypertension and in human patients with POAG, demonstrating an increase in ECM deposition in the TM [2–4, 42, 56]. It is believed that the inner wall of SC and the JCT are the primary location for aqueous humor outflow resistance [2, 3]. The presence of ECM in the TM and JCT region is necessary to maintain the outflow as it creates space between cells and trabecular lamina, and the ECM is also bound to proteoglycans which have been shown to be necessary for efficient outflow [5]. There are several types of ECM in the TM which are constantly secreted, maintained, and recycled by TM cells. However, with aging and/or disease the normal balance of ECM production and degradation appears to be disrupted, leading to increased resistance to outflow and elevated IOP. In addition, it is known that in POAG the connecting fibrils between the JCT TM and SC inner wall endothelium are thickened forming sheath-derived plaques; and the basement membrane material underlying SC endothelium is thicker and more continuous [7]. Abnormal accumulation of ECM material in the TM and JCT region has been shown in several histological analyzes of POAG eyes [4, 8, 23, 57–59]. Amorphous ECM material in the JCT region is made up of primarily collagen type IV, laminin, and fibronectin [60]. The ECM material and sheath-derived plaques in the JCT region and on the inner side of SC endothelium is increased in glaucomatous eyes [4, 7, 61]. These data are recapitulated here in our B6.EDA^+/+^ mouse model where we have identified an increase in FN protein expression by immunohistochemistry and total ECM accumulation by electron microscopy.

Aqueous humor is transported across the inner wall endothelium of SC through tight junctions of SC cells, as well as through the formation of giant vacuoles and associated pores in SC endothelial cells [62–64]. The cavity of giant vacuoles are entirely extracellular and giant vacuoles are described as outpouchings of the SC endothelial cells themselves. Giant vacuoles bulge into the lumen of SC with the aqueous humor filled cavity remaining between the cell and the underlying basement membrane [65, 66]. The thicker and more continuous SC basement membrane and the increase in ECM deposition prevents aqueous humor from efficiently crossing into SC. These data are supported by recent work showing increased stiffness and flow resistance at the inner wall of SC [2]. Early studies by Tripathi et al. showed a quantitative and qualitative depletion of giant vacuoles in glaucoma [61]. In addition, previous histological studies have demonstrated a decrease in the number of pores in eyes with POAG [67, 68]. However, similar giant vacuole size and density was observed in a recent study of ex vivo perfused human POAG eyes, even though the POAG eyes had a lower flow rate due to a higher flow resistance [2]. Consistent with these results, we show similar giant vacuole numbers between control and B6.EDA^+/+^ mice, but B6.EDA^+/+^ do have significantly more smaller giant vacuoles. However, we did not analyze the number of number of giant vacuoles with pores in the B6.EDA^+/+^ mice.

In addition to changes in the ECM of the TM, we also observed a significant increase in Iba-1 positive cells in B6.EDA^+/+^ mice compared to controls. Iba1 is both specifically expressed and upregulated in the activation of macrophages/microglia. Macrophages are known to be present in the TM [69, 70] and there is some evidence that macrophage inflammatory proteins are differentially expressed in the TM from POAG patients [71]. Previous studies also suggest that during glaucoma macrophages produce cytokines such as IL6, IL1β and TNFα that could lead to an acute inflammatory response [71, 72]. Although the existing literature suggests macrophages are present in the TM and may produce disease-relevant signaling molecules, a role for macrophages in IOP regulation and ocular hypertension has not been identified. Here, we identify that Iba-1 positive cells are expressed and upregulated in ocular hypertensive B6.EDA^+/+^ mice, recapitulating what has been shown in the TM of POAG patients [71]. These data suggest that B6.EDA^+/+^ mice are an excellent model system to further study the role of macrophages and inflammatory proteins in glaucoma.

We have previously identified that EDA-induced ocular hypertension and EDA-induced ECM production is dependent on TLR4 [13, 24]. We also identified that TGFβ2-induced ocular hypertension is dependent on both EDA and TLR4 [13, 24]. In addition, TLR4 is known to be expressed in the TM [13, 73]. Here, we demonstrate by RNAscope in situ hybridization that *Tlr4* is expressed in the mouse TM and increased in the TM of B6.EDA^+/+^ mice compared to controls, similar to prior studies which indicate that TLR4 activation can stimulate increased expression of TLR4 [27]. These data highlight the importance of EDA activated TLR4 in the regulation of ECM and ocular hypertension. In addition, we also demonstrate the expression of *Tlr4* in mouse ONH astrocytes, microglia, and RGC axons by RNAScope in situ hybridization. There are several conflicting reports on whether *Tlr4* is expressed in rodent astrocytes; however, these studies involved astrocytes isolated from brain which could indicate tissue specific expression [74–77]. Consistent with our findings, Gorina et al. reported expression of *Tlr4* in mouse astrocytes [78]. In addition, neuroinflammatory responses involving astrocytes and microglia are recognized in the ONH in glaucoma, and evidence from both human glaucoma patients and animal models of glaucoma suggest that immune responses are mediated, at least in part, by TLR4 [79–81]. These data suggest that mice are a good model to study human relevant disease pathology in astrocytes of the retina and optic nerve in the context of neuroinflammatory processes involved in the development of glaucoma. Further analysis of the ONH cells and tissue will determine the role of TLR4 and FN-EDA in the development of glaucomatous damage.

In POAG, elevated IOP is followed by progressive damage to the ONH and loss of RGCs. Previously it has been reported that microglial activation is also associated with early stages of experimental glaucoma [46, 82–85], and the extent of microglial activation is closely related to axonal degeneration [44, 47]. Increased expression of Iba1 has previously been used as a measure of microglial activation in the ONH and other parts of the CNS [46, 86]. Here we show that Iba1 expression is increased in the ONH of B6.EDA^+/+^ mice compared to controls at 8–9 months of age. Subsequently, at 12 months of age we show mild ON damage and loss of RGCs. We utilized a well-established ON grading scheme [41] of p-phenylenediamine (PPD) stained cross-sections of ONs. Based on the extent of staining, masked observers assign a score of 1–5 to each section, with a score of 1 indicating very few dark staining axons, no identifiable gliosis or axonal swelling. A score of 5 indicates less than 10% of axons alive and gliosis and darkly stained axons making up 95% of the ON. Here, many of the ONs in B6.EDA^+/+^ mice scored 2 on the grading scale, indicative of 5–10% darkly stained axons and the initial stage of gliosis. Although this type of grading scheme is semiquantitative in nature, it has previously been found to correlate well with RGC density and axon density in the ON [87, 88]. Indeed, we found a correlative loss of 9.7% of RGC axons at 12 months of age in B6.EDA^+/+^ mice. In summary, these data show the development of slowly progressive glaucomatous damage in B6.EDA^+/+^ mice.

## Conclusions

Our results show that B6.EDA^+/+^ mice have ultrastructural changes to the TM consistent with POAG. We also demonstrate the differential expression of key fibrotic pathway components, FN, FN-EDA, and TLR4 in the TM of B6.EDA^+/+^ mice. In addition, we described early microglial activation and subsequent glaucomatous damage to the retina and ON. In summary, B6.EDA + / + mice harbor many phenotypes of glaucoma and provide an excellent resource to study the molecular pathology of the disease.

## Methods

### Animals

All experiments were conducted in compliance with the ARVO Statement for the Use of Animals in Ophthalmic and Vision Research and approved by the University of Wisconsin-Madison Institutional Animal Care and Use Committee (IACUC) Guidelines and Regulations. The generation of B6.EDA^−/−^ and B6.EDA^+/+^ mice has previously been described [89]. B6.EDA^+/+^ mice were generated to contain spliced sites at both splicing junctions of the EDA exon and therefore constitutively express only FN containing EDA. B6.EDA^−/−^ mice contain an EDA-null allele of the EDA exon and express only FN lacking EDA. C57BL/6J mice were purchased from the Jackson Laboratories. All animals were housed in the UW-Madison vivarium. All mice were maintained on a normal 12-h light/dark cycle and maintained on a 4% fat diet (Harkland Teklad, Madison, WI, USA) with food and water available ad libitum.

### Ultrastructural analysis

For ultrastructural analysis mice were euthanized by CO2 inhalation, eyes were enucleated, placed in 0.1 M Sodium cacodylate buffer, and a small puncture was made in the cornea for the immediate access of fixative to the TM tissue. Eyes were then transferred into fixative (2.5% Paraformaldehyde, 2.5% Glutaraldehyde in 0.1 M cacodylate buffer) and incubated at room temperature overnight on a rotator. Eyes were postfixed in a mixture of 1% OsO4, 0.8% potassium ferrocyanide in 0.1 M cacodylate buffer for 3.5 h at room temperature. Samples were rinsed in cacodylate buffer, followed by water, dehydrated in graded series of 35%, 50%, 60%, 70% ethanol, each time for 15 min twice. Next, samples were stained in 2% uranyl acetate in 70% ethanol for 2 h, rinsed in 70% ethanol overnight, followed by 80%, 90%, 95%,100% ethanol each time for 15 min twice. Samples were further dehydrated in mixture of 1/1 Ethanol/Acetone, followed by two rinses in 100% acetone for 15 min each. Further, samples were embedded into graded mixtures of 3/1, 1/1, 1/3 Acetone/EPON for 12 h each, followed by two changes of pure EPON for 24 h each time. To remove residual water, samples were placed into a vacuum for 1 h, and polymerized in EPON at 60 degrees Celsius for 24 h. Following polymerization, 80 nm thin ultrathin sections were cut, placed on formvar coated slot grids, and then poststained with uranyl acetate and lead citrate. Images were taken using a Philips Electron Microscope. From anterior to posterior, consecutive images of the entire length of the TM and SC area were collected at × 2500. For measurement of total ECM, images were stitched together in ImageJ using the “pairwise stitching” plugin. The Trainable Weka Segmentation plugin was utilized to create a selection of the area occupied by ECM. For this, a specific classifier was created by drawing lines over the ECM, and separately over non-ECM structures (including empty spaces) to create the selected area and, ultimately, to mask the selection. The entire area of the TM was masked in ImageJ with the polygonal selection tool, and pixel area of TM was calculated, equating to the area occupied by ECM. In total, three animals from each genotype were evaluated, with two planes of stitched sections along the entire length of SC analyzed per mouse. For measurement of the length of basement membrane material (BMM) and size and density of giant vacuoles, images of the entire length of SC were collected at 12,000× and stitched with plugging “pairwise stitching” in ImageJ as described above. Area of vacuoles was measured in ImageJ with polygonal tool selection, and length of BMM was measured with “segmented line” selection.

### Immunocytochemistry

Animals were euthanized by exposure to CO_2_. Eyes were enucleated, and immediately transferred into 4% PFA in PBS and fixed overnight. Eyes were rinsed, dehydrated in increasing concentrations of ethanol, and embedded into paraffin. Five micron thick sections were cut. Sections were deparaffinized in two incubations of xylenes for 2 min each and then rinsed twice in 100% ethanol, 95% ethanol, and water, 2 min each. Antigen retrieval was performed in Sodium Citrate Buffer for 10 min at 95 degrees Celsius. Sections were incubated in Superblock for one hour and stained with primary antibody (see Table 1 list of antibodies) in Superblock overnight. Sections were rinsed trice in PBS, 10 min each time, and stained for two hours with appropriate fluorescently conjugated secondary antibodies. Finally, sections were rinsed, mounted into Prolong Diamond Antifade with DAPI (ThermoFisher #P36971) or without DAPI where applicable (ThermoFisher #P36970), and coverslipped. Images were acquired with a Nikon A2 laser confocal microscope (Nikon, Tokyo, Japan) using NIS elements software. Acquired Z-stacks were analyzed with ImageJ for a single plane selection.

**Table 1:** Primary and secondary antibodies used for all experiments.

Antibody	Raised in Species	Company, Catalogue#	Application	Dilution
Primary antibodies				
TLR4	Rabbit	Proteintech, #19811-1AP	WB	1/1000
Iba-1	Rabbit	Wako, #019-19741	IHC	1/1000
Fibronectin	Rabbit	Millipore, AB1945	IHC	1/500
GAPDH	Mouse	Cell Signalling, #97166	WB	1/5000
NFH	Mouse	Biolegend, #801701	IHC	1/500
NFH	Mouse	Biolegend, #835503	IHC	1/500
GFAP	Chicken	AVES, #AVES-GFAP	IHC	1/500
RBPMS	Guinea Pig	Phosphosolutions, #1832RBPMS	IHC	1/200
Secondary antibodies				
Anti-Chicken 680	Donkey	LI-COR, 925-68075	WB	1/10000
Anti-Mouse 800	Goat	LI-COR, 827-08364	WB	1/10000
Anti-Rabbit 800	Goat	LI-COR, 925-68071	WB	1/10000
Anti-Mouse 680	Goat	LI-COR, 926-68170	WB	1/10000
Anti-Rabbit Cy2	Donkey	Jackson Immunoresearch, #711225152	IHC	1/500
Anti-Mouse Cy2	Donkey	Jackson Immunoresearch, #715225150	IHC	1/500
Anti-Rabbit Cy2	Donkey	Jackson Immunoresearch, #71175152	IHC	1/500
Anti-Guinea Pig Cy3	Donkey	Jackson Immunoresearch #706165148	IHC	1/500

### In Situ hybridization

To detect *Tlr4* mRNA mouse mRNA probes (#316,801) were purchased from ACD company. Incubation and detection of probes was done according to manufacture instructions. Briefly, frozen sections (in OCT) on slides were baked at 60 degrees Celsius for 30 min, pretreated with hydrogen peroxide. Antigen retrieval was performed in Antigen Retrieval buffer for 5 min at 95 degrees Celsius. Slides were laid flat during the antigen retrieval step to diminish the chance of the section detaching. Hydrophobic borders were created around sections with ImmEdge pen (#H-4000, Vector Laboratories), and proteinase K applied for 30 min at 40 degrees Celsius, all performed in a humidified chamber. Sections were rinsed and the mRNA probe was added for 2 h, followed by a series of rinses and addition of amplification reagents each for 30 min. Signal was amplified with TSA Cy3 reagent (Akoya #SAT704A001EA). After in situ hybridization was accomplished, sections were additionally stained with antibodies against various targets (as described above) using fluorophore conjugated secondary antibody selected to avoid interference with RNA detection signal.

### Image analysis

For image analysis of the percent of area occupied by specific cellular markers detected in confocal images of the ONH (IBA-1 for microglia, NF-200 for GC axons, GFAP for astrocytes), the total area of the ONH was calculated in ImageJ using the polygonal selection tool. Individual channels were separated, and a threshold mask (same threshold values in all conditions) was applied for individual channels, and threshold area of each channel was calculated. Percent of occupancy by individual threshold area of each channel inside of outlined ONH area was calculated in ImageJ. Similar analysis was performed for total FN and Iba1 labeling in the TM.

For quantification of numbers of mRNA particles in the TM and ONH, automatic counting was performed using ImageJ. Binary images of mRNA puncta were created by using a threshold (same values for all images) and creating a selection of it (Edit/Create Selection command) followed by Watershed processing function (Process/Binary/Watershed) to prevent counting cluster of puncta as a single value. Particles with a size less than two pixels were assigned as noise rather than signal and were not included in counting. Finally, puncta were counted and calculated per 100 square microns of masked area of TM.

### Western blotting

Primary mouse astrocytes (passage 11) were isolated from mouse ONH as we previously described [90], and were grown to confluency in 6-well plates. Cells were rinsed with DPBS, and RIPA buffer (200 µl/well) supplemented with protease/phosphatase cocktail inhibitors (MilliporeSigma #11,836,170,001; #4,906,845,001) was added for 10 min on ice with gentle rocking. The cell extract was collected and centrifuged at 14000×*g* for 10 min and protein concentration measured by BCA assay. Samples were loaded onto a polyacrylamide gel (25 µg/well) and resolved with constant voltage at 200 V for 45 min. Resolved samples from the gel were transferred to PVDF and blocked in the Intercept protein-free blocking buffer (LI-COR, #927–65,001) for 1 h at room temperature. Primary antibody (see Table 1) was added in the incubation buffer (same blocking buffer with addition of 0.2% Tween) and incubated overnight at 4 degrees Celsius. Secondary antibody was applied for 1 hour in incubation buffer at room temperature. Fluorescent signal was detected using the Odyssey Licor System.

### Intraocular pressure measurements

 IOP was measured with a rebound tonometer as we previously described [13, 24]. Mice were acclimated to the procedure room, and IOP was measured using a non-invasive method in isoflurane anesthetized mice with the TonoLab tonometer (Colonial Medical Supply, Franconia, NH). All measurements were made during the same 2-h period of the lights-on phase. All IOP’s are represented as means (+ / − SEM) and statistical significance determined by two-tailed paired Student’s t-test, n = 16–22 eyes/strain.

### Quantification of retina ganglion cell loss

RGC loss was quantified in RPBMS labeled retina flat mounts and quantified by counting RBMPS positive cells in 4 central and 4 peripheral regions of the retina as previously described [91, 92]. Dissected retinas were pre-treated in 0.3% TritonX-100 in PBS for 30 min (× 4) and then blocked in 0.3% Triton X-100 in PBS containing 10% goat serum for 2 h. RGCs were labeled using anti-RBPMS antibody overnight at 4 °C. Following washes in PBS, the retinas were incubated with Donkey anti-Guinea Pig Cy3 conjugated antibody (diluted in PBST) overnight at 4 °C and mounted with Vectashield Mounting Medium containing DAPI (Vector Laboratories #H-1200–10) and coverslipped. Images were collected using a epifluorescent Zeiss Axiovert microscope. Z-stacks (within a thickness of 50 microns, 5 microns per optical Z-slice) were collected of the entire mouse flat retinas with the central Z-focus on RGC layer to include all RGCs throughout the tiles of the entire retina. Tiles were automatically stitched in Zen Blue software. Images were opened in ImageJ, composites of Z-stacks were created, and total eight quadrants (0.25 × 0.25 mm) were selected at the distance 0.7 mm (central region) and 1.4 mm (peripheral region) away from the ON. Cells were manually counted in ImageJ in a masked manner using the multi-point tool. For each individual retina, the RGC count was obtained by averaging the eight fields for each retina and calculating average RGC number per square millimeter. Significance was determined by two-tailed paired Student’s t-test, n = 10–14 eyes/strain.

### Quantification of optic nerve damage

ON damage was assessed in sections of resin embedded ON’s by paraphenylenediamine (PPD) staining and ranked for damage using an ON-damage score scale by two individuals in a masked manner as we previously described [34, 41]. Retro-orbital optic nerves were fixed, processed, and embedded in plastic. Optic nerve cross-sections were stained with PPD which stains the myelin sheaths and more darkly stains the axoplasm of sick or dying axons. We and others have previously quantified optic nerve damage successfully using semi-quantitative optic nerve grading schemes [34, 87, 88, 93]. Here, we used a five-point optic nerve grading scheme [41]. Two investigators performed masked evaluations to grade each optic nerve, and optic nerve damage scores for each sample were averaged. Statistical significance was determined by Fisher's exact test, n = 18–23 ONs/strain.

## Supplementary Information

**Additional file 1: Figure S1.** Mouse Tlr4 mRNA probe is specific. **A**, **B** In situ hybridization of *Tlr4* in C57BL/6J controls confirms expression of *Tlr4* in the mouse ONH, while no specific labeling was detected in *Tlr4* knock out (TLR4^-/-^) mice.

## Data Availability

All data generated or analyzed during this study are included in this published article [and its Additional information files] and are available from the corresponding author on reasonable request.

## Abbreviations

FN: Fibronectin
FN-EDA: Fibronectin extra domain A
Tlr4: Toll like receptor 4
TM: Trabecular meshwork
SC: Schlemm’s canal
JCT: Juxtacanalicular tissue
POAG: Primary open angle glaucoma
IOP: Intraocular pressure
AH: Aqueous humor
GV: Giant vacuoles
BMM: Basement membrane material

## References

[1] Tamm ER (2009). The trabecular meshwork outflow pathways: structural and functional aspects. Exp Eye Res.

[2] Vahabikashi A, Gelman A, Dong B, Gong L, Cha EDK, Schimmel M (2019). Increased stifness and flow resistance of the inner wall of Schlemm's canal in glaucomatous human eyes. Proc Natl Acad Sci USA.

[3] Lutjen-Drecoll E (1999). Functional morphology of the trabecular meshwork in primate eyes. Prog Retin Eye Res.

[4] Rohen JW, Witmer R (1972). Electrn microscopic studies on the trabecular meshwork in glaucoma simplex. Albrecht Von Graefes Arch Klin Exp Ophthalmol.

[5] Keller KE, Aga M, Bradley JM, Kelley MJ, Acott TS (2009). Extracellular matrix turnover and outflow resistance. Exp Eye Res.

[6] Babizhayev MA, Brodskaya MW (1993). Immunohistochemical monitoring of the effect of a synthetic fibronectin-like peptide (Arg-Gly-Asp) on the age-related changes in the isolated human corneoscleral tissue of glaucomatous eyes. Mech Ageing Dev.

[7] Lutjen-Drecoll E, Shimizu T, Rohrbach M, Rohen JW (1986). Quantitative analysis of 'plaque material' in the inner- and outer wall of Schlemm's canal in normal- and glaucomatous eyes. Exp Eye Res.

[8] Rohen JW, Lutjen-Drecoll E, Flugel C, Meyer M, Grierson I (1993). Ultrastructure of the trabecular meshwork in untreated cases of primary open-angle glaucoma (POAG). Exp Eye Res.

[9] Knepper PA, Goossens W, Hvizd M, Palmberg PF (1996). Glycosaminoglycans of the human trabecular meshwork in primary open-angle glaucoma. Invest Ophthalmol Vis Sci.

[10] Russell P, Johnson M (2012). Elastic modulus determination of normal and glaucomatous human trabecular meshwork. Invest Ophthalmol Vis Sci.

[11] Vranka JA, Staverosky JA, Reddy AP, Wilmarth PA, David LL, Acott TS (2018). Biomechanical rigidity and quantitative proteomics analysis of segmental regions of the trabecular meshwork at physiologic and elevated pressures. Invest Ophthalmol Vis Sci.

[12] Acott TS, Kelley MJ (2008). Extracellular matrix in the trabecular meshwork. Exp Eye Res.

[13] Hernandez H, Medina-Ortiz WE, Luan T, Clark AF, McDowell CM (2017). Crosstalk between transforming growth factor beta-2 and toll-like receptor 4 in the trabecular meshwork. Invest Ophthalmol Vis Sci.

[14] Wordinger RJ, Fleenor DL, Hellberg PE, Pang IH, Tovar TO, Zode GS (2007). Effects of TGF-beta2, BMP-4, and gremlin in the trabecular meshwork: implications for glaucoma. Invest Ophthalmol Vis Sci.

[15] Faralli JA, Schwinn MK, Gonzalez JM, Filla MS, Peters DM (2009). Functional properties of fibronectin in the trabecular meshwork. Exp Eye Res.

[16] White ES, Baralle FE, Muro AF (2008). New insights into form and function of fibronectin splice variants. J Pathol.

[17] Ffrench-Constant C (1995). Alternative splicing of fibronectin–many different proteins but few different functions. Exp Cell Res.

[18] Serini G, Bochaton-Piallat ML, Ropraz P, Geinoz A, Borsi L, Zardi L (1998). The fibronectin domain ED-A is crucial for myofibroblastic phenotype induction by transforming growth factor-beta1. J Cell Biol.

[19] Muro AF, Chauhan AK, Gajovic S, Iaconcig A, Porro F, Stanta G (2003). Regulated splicing of the fibronectin EDA exon is essential for proper skin wound healing and normal lifespan. J Cell Biol.

[20] Kuhn C, Boldt J, King TE, Crouch E, Vartio T, McDonald JA (1989). An immunohistochemical study of architectural remodeling and connective tissue synthesis in pulmonary fibrosis. Am Rev Respir Dis.

[21] Ffrench-Constant C, Van de Water L, Dvorak HF, Hynes RO (1989). Reappearance of an embryonic pattern of fibronectin splicing during wound healing in the adult rat. J Cell Biol.

[22] Hino K, Shiozawa S, Kuroki Y, Ishikawa H, Shiozawa K, Sekiguchi K (1995). EDA-containing fibronectin is synthesized from rheumatoid synovial fibroblast-like cells. Arthritis Rheum.

[23] Medina-Ortiz WE, Belmares R, Neubauer S, Wordinger RJ, Clark AF (2013). Cellular fibronectin expression in human trabecular meshwork and induction by transforming growth factor-beta2. Invest Ophthalmol Vis Sci.

[24] Roberts AL, Mavlyutov TA, Perlmutter TE, Curry SM, Harris SL, Chauhan AK (2020). Fibronectin extra domain A (FN-EDA) elevates intraocular pressure through Toll-like receptor 4 signaling. Sci Rep.

[25] Sharma TP, Curry S, McDowell CM (2020). Effects of toll-like receptor 4 inhibition on transforming growth factor-beta2 signaling in the human trabecular meshwork. J Ocul Pharmacol Ther.

[26] Okamura Y, Watari M, Jerud ES, Young DW, Ishizaka ST, Rose J (2001). The extra domain A of fibronectin activates Toll-like receptor 4. J Biol Chem.

[27] Bhattacharyya S, Kelley K, Melichian DS, Tamaki Z, Fang F, Su Y (2013). Toll-like receptor 4 signaling augments transforming growth factor-beta responses: a novel mechanism for maintaining and amplifying fibrosis in scleroderma. Am J Pathol.

[28] Seki E, De Minicis S, Osterreicher CH, Kluwe J, Osawa Y, Brenner DA (2007). TLR4 enhances TGF-beta signaling and hepatic fibrosis. Nat Med.

[29] Pulskens WP, Rampanelli E, Teske GJ, Butter LM, Claessen N, Luirink IK (2010). TLR4 promotes fibrosis but attenuates tubular damage in progressive renal injury. J Am Soc Nephrol.

[30] Campbell MT, Hile KL, Zhang H, Asanuma H, Vanderbrink BA, Rink RR (2011). Toll-like receptor 4: a novel signaling pathway during renal fibrogenesis. J Surg Res.

[31] Pang IH, Clark AF (2019). Inducible rodent models of glaucoma. Prog Retin Eye Res.

[32] Hernandez H, Millar JC, Curry SM, Clark AF, McDowell CM (2018). BMP and activin membrane bound inhibitor regulates the extracellular matrix in the trabecular meshwork. Invest Ophthalmol Vis Sci.

[33] McDowell CM, Hernandez H, Mao W, Clark AF (2015). Gremlin induces ocular hypertension in mice through smad3-dependent signaling. Invest Ophthalmol Vis Sci.

[34] McDowell CM, Luan T, Zhang Z, Putliwala T, Wordinger RJ, Millar JC (2012). Mutant human myocilin induces strain specific differences in ocular hypertension and optic nerve damage in mice. Exp Eye Res.

[35] Shepard AR, Millar JC, Pang IH, Jacobson N, Wang WH, Clark AF (2010). Adenoviral gene transfer of active human transforming growth factor-{beta}2 elevates intraocular pressure and reduces outflow facility in rodent eyes. Invest Ophthalmol Vis Sci.

[36] Junglas B, Kuespert S, Seleem AA, Struller T, Ullmann S, Bosl M (2012). Connective tissue growth factor causes glaucoma by modifying the actin cytoskeleton of the trabecular meshwork. Am J Pathol.

[37] Wang WH, McNatt LG, Pang IH, Millar JC, Hellberg PE, Hellberg MH (2008). Increased expression of the WNT antagonist sFRP-1 in glaucoma elevates intraocular pressure. J Clin Investig.

[38] Mao W, Millar JC, Wang WH, Silverman SM, Liu Y, Wordinger RJ (2012). Existence of the canonical Wnt signaling pathway in the human trabecular meshwork. Invest Ophthalmol Vis Sci.

[39] Pang IH, Millar JC, Clark AF (2015). Elevation of intraocular pressure in rodents using viral vectors targeting the trabecular meshwork. Exp Eye Res.

[40] McDowell CM, Kizhatil K, Elliott MH, Overby DR, van Batenburg-Sherwood J, Millar JC (2022). Consensus recommendation for mouse models of ocular hypertension to study aqueous humor outflow and its mechanisms. Invest Ophthalmol Vis Sci.

[41] Pang IH, Clark AF (2007). Rodent models for glaucoma retinopathy and optic neuropathy. J Glaucoma.

[42] Overby DR, Bertrand J, Tektas OY, Boussommier-Calleja A, Schicht M, Ethier CR (2014). Ultrastructural changes associated with dexamethasone-induced ocular hypertension in mice. Invest Ophthalmol Vis Sci.

[43] Tamm ER, Braunger BM, Fuchshofer R (2015). Intraocular pressure and the mechanisms involved in resistance of the aqueous humor flow in the trabecular meshwork outflow pathways. Prog Mol Biol Transl Sci.

[44] Bosco A, Crish SD, Steele MR, Romero CO, Inman DM, Horner PJ (2012). Early reduction of microglia activation by irradiation in a model of chronic glaucoma. PLoS ONE.

[45] Bosco A, Romero CO, Breen KT, Chagovetz AA, Steele MR, Ambati BK (2015). Neurodegeneration severity can be predicted from early microglia alterations monitored in vivo in a mouse model of chronic glaucoma. Dis Model Mech.

[46] Bosco A, Steele MR, Vetter ML (2011). Early microglia activation in a mouse model of chronic glaucoma. J Comp Neurol.

[47] Howell GR, Soto I, Zhu X, Ryan M, Macalinao DG, Sousa GL (2012). Radiation treatment inhibits monocyte entry into the optic nerve head and prevents neuronal damage in a mouse model of glaucoma. J Clin Investig.

[48] Ito D, Imai Y, Ohsawa K, Nakajima K, Fukuuchi Y, Kohsaka S (1998). Microglia-specific localisation of a novel calcium binding protein, Iba1. Brain Res Mol Brain Res.

[49] Emes RD, Goodstadt L, Winter EE, Ponting CP (2003). Comparison of the genomes of human and mouse lays the foundation of genome zoology. Hum Mol Genet.

[50] Huang H, Winter EE, Wang H, Weinstock KG, Xing H, Goodstadt L (2004). Evolutionary conservation and selection of human disease gene orthologs in the rat and mouse genomes. Genome Biol.

[51] May CA, Lutjen-Drecoll E (2002). Morphology of the murine optic nerve. Invest Ophthalmol Vis Sci.

[52] Smith RS (2002). Systemic evaluation of the mouse eye.

[53] Lei Y, Overby DR, Boussommier-Calleja A, Stamer WD, Ethier CR (2011). Outflow physiology of the mouse eye: pressure dependence and washout. Invest Ophthalmol Vis Sci.

[54] Overby DR, Bertrand J, Schicht M, Paulsen F, Stamer WD, Lutjen-Drecoll E (2014). The structure of the trabecular meshwork, its connections to the ciliary muscle, and the effect of pilocarpine on outflow facility in mice. Invest Ophthalmol Vis Sci.

[55] Boussommier-Calleja A, Bertrand J, Woodward DF, Ethier CR, Stamer WD, Overby DR (2012). Pharmacologic manipulation of conventional outflow facility in ex vivo mouse eyes. Invest Ophthalmol Vis Sci.

[56] Wang K, Li G, Read AT, Navarro I, Mitra AK, Stamer WD (2018). The relationship between outflow resistance and trabecular meshwork stiffness in mice. Sci Rep.

[57] Lutjen-Drecoll E, Futa R, Rohen JW (1981). Ultrahistochemical studies on tangential sections of the trabecular meshwork in normal and glaucomatous eyes. Invest Ophthalmol Vis Sci.

[58] Rohen JW, Futa R, Lutjen-Drecoll E (1981). The fine structure of the cribriform meshwork in normal and glaucomatous eyes as seen in tangential sections. Invest Ophthalmol Vis Sci.

[59] Alvarado JA, Yun AJ, Murphy CG (1986). Juxtacanalicular tissue in primary open angle glaucoma and in nonglaucomatous normals. Arch Ophthalmol.

[60] Ueda J, Wentz-Hunter K, Yue BY (2002). Distribution of myocilin and extracellular matrix components in the juxtacanalicular tissue of human eyes. Invest Ophthalmol Vis Sci.

[61] Tripathi RC (1972). Aqueous outflow pathway in normal and glaucomatous eyes. Br J Ophthalmol.

[62] Grierson I, Lee WR (1975). Pressure-induced changes in the ultrastructure of the endothelium lining Schlemm's canal. Am J Ophthalmol.

[63] Lee WR, Grierson I (1975). Pressure effects on the endothelium of the trabecular wall of Schlemm's canal: a study by scanning electron microscopy. Albrecht Von Graefes Arch Klin Exp Ophthalmol.

[64] Tripathi RC (1968). Ultrastructure of Schlemm's canal in relation to aqueous outflow. Exp Eye Res.

[65] Garron LK, Feeney ML, Hogan MJ, Mcas EW (1958). Electron microscopic studies of the human eye. I. Preliminary investigations of the trabeculas. Am J Ophthalmol.

[66] Pedrigi RM, Simon D, Reed A, Stamer WD, Overby DR (2011). A model of giant vacuole dynamics in human Schlemm's canal endothelial cells. Exp Eye Res.

[67] Allingham RR, de Kater AW, Ethier CR, Anderson PJ, Hertzmark E, Epstein DL (1992). The relationship between pore density and outflow facility in human eyes. Invest Ophthalmol Vis Sci.

[68] Johnson M, Chan D, Read AT, Christensen C, Sit A, Ethier CR (2002). The pore density in the inner wall endothelium of Schlemm's canal of glaucomatous eyes. Invest Ophthalmol Vis Sci.

[69] McMenamin PG, Holthouse I (1992). Immunohistochemical characterization of dendritic cells and macrophages in the aqueous outflow pathways of the rat eye. Exp Eye Res.

[70] Patel G, Fury W, Yang H, Gomez-Caraballo M, Bai Y, Yang T (2020). Molecular taxonomy of human ocular outflow tissues defined by single-cell transcriptomics. Proc Natl Acad Sci USA.

[71] Micera A, Quaranta L, Esposito G, Floriani I, Pocobelli A, Sacca SC (2016). Differential protein expression profiles in glaucomatous trabecular meshwork: an evaluation study on a small primary open angle glaucoma population. Adv Ther.

[72] Taurone S, Ripandelli G, Pacella E, Bianchi E, Plateroti AM, De Vito S (2015). Potential regulatory molecules in the human trabecular meshwork of patients with glaucoma: immunohistochemical profile of a number of inflammatory cytokines. Mol Med Rep.

[73] Grybauskas A, Koga T, Kuprys PV, Nolan M, McCarty R, Walker L (2015). ABCB1 transporter and Toll-like receptor 4 in trabecular meshwork cells. Mol Vis.

[74] Cahoy JD, Emery B, Kaushal A, Foo LC, Zamanian JL, Christopherson KS (2008). A transcriptome database for astrocytes, neurons, and oligodendrocytes: a new resource for understanding brain development and function. J Neurosci: The Official Journal of the Society for Neuroscience.

[75] Liddelow SA, Guttenplan KA, Clarke LE, Bennett FC, Bohlen CJ, Schirmer L (2017). Neurotoxic reactive astrocytes are induced by activated microglia. Nature.

[76] Zhang Y, Chen K, Sloan SA, Bennett ML, Scholze AR, O'Keeffe S (2014). An RNA-sequencing transcriptome and splicing database of glia, neurons, and vascular cells of the cerebral cortex. J Neurosci: The Official Journal of the Society for Neuroscience.

[77] Zhang Y, Sloan SA, Clarke LE, Caneda C, Plaza CA, Blumenthal PD (2016). Purification and characterization of progenitor and mature human astrocytes reveals transcriptional and functional differences with mouse. Neuron.

[78] Gorina R, Font-Nieves M, Marquez-Kisinousky L, Santalucia T, Planas AM (2011). Astrocyte TLR4 activation induces a proinflammatory environment through the interplay between MyD88-dependent NFkappaB signaling, MAPK, and Jak1/Stat1 pathways. Glia.

[79] Luo C, Yang X, Kain AD, Powell DW, Kuehn MH, Tezel G (2010). Glaucomatous tissue stress and the regulation of immune response through glial Toll-like receptor signaling. Invest Ophthalmol Vis Sci.

[80] Howell GR, Macalinao DG, Sousa GL, Walden M, Soto I, Kneeland SC (2011). Molecular clustering identifies complement and endothelin induction as early events in a mouse model of glaucoma. J Clin Investig.

[81] Howell GR, Walton DO, King BL, Libby RT, John SW (2011). Datgan, a reusable software system for facile interrogation and visualization of complex transcription profiling data. BMC Genomics.

[82] Neufeld AH (1999). Microglia in the optic nerve head and the region of parapapillary chorioretinal atrophy in glaucoma. Arch Ophthalmol.

[83] Yuan L, Neufeld AH (2001). Activated microglia in the human glaucomatous optic nerve head. J Neurosci Res.

[84] Ebneter A, Casson RJ, Wood JP, Chidlow G (2010). Microglial activation in the visual pathway in experimental glaucoma: spatiotemporal characterization and correlation with axonal injury. Invest Ophthalmol Vis Sci.

[85] Johnson EC, Morrison JC (2009). Friend or foe? Resolving the impact of glial responses in glaucoma. J Glaucoma.

[86] Nilsson I, Lindfors C, Fetissov SO, Hokfelt T, Johansen JE (2008). Aberrant agouti-related protein system in the hypothalamus of the anx/anx mouse is associated with activation of microglia. J Comp Neurol.

[87] Anderson MG, Libby RT, Gould DB, Smith RS, John SW (2005). High-dose radiation with bone marrow transfer prevents neurodegeneration in an inherited glaucoma. Proc Natl Acad Sci USA.

[88] Chauhan BC, Levatte TL, Garnier KL, Tremblay F, Pang IH, Clark AF (2006). Semiquantitative optic nerve grading scheme for determining axonal loss in experimental optic neuropathy. Invest Ophthalmol Vis Sci.

[89] Muro AF, Chauhan AK, Gajovic S, Iaconcig A, Porro F, Stanta G (2003). Regulated splicing of the fibronectin EDA exon is essential for proper skin wound healing and normal lifespan. J Cell Biol.

[90] Liu Y, Patel GC, Mao W, Clark AF (2018). Establishment of a conditionally immortalized mouse optic nerve astrocyte line. Exp Eye Res.

[91] Daniel S, Clark AF, McDowell CM (2018). Subtype-specific response of retinal ganglion cells to optic nerve crush. Cell Death Discov.

[92] Daniel S, Meyer KJ, Clark AF, Anderson MG, McDowell CM (2019). Effect of ocular hypertension on the pattern of retinal ganglion cell subtype loss in a mouse model of early-onset glaucoma. Exp Eye Res.

[93] Libby RT, Anderson MG, Pang IH, Robinson ZH, Savinova OV, Cosma IM (2005). Inherited glaucoma in DBA/2J mice: pertinent disease features for studying the neurodegeneration. Vis Neurosci.

