# Supplementary figures and images for: Fibronectin extra domain A (FN-EDA) causes glaucomatous trabecular meshwork, retina, and optic nerve damage in mice
Source: Cell Biosci. 2022 May 26;12:72. doi: 10.1186/s13578-022-00800-y (PMC9137085; doi:10.1186/s13578-022-00800-y)

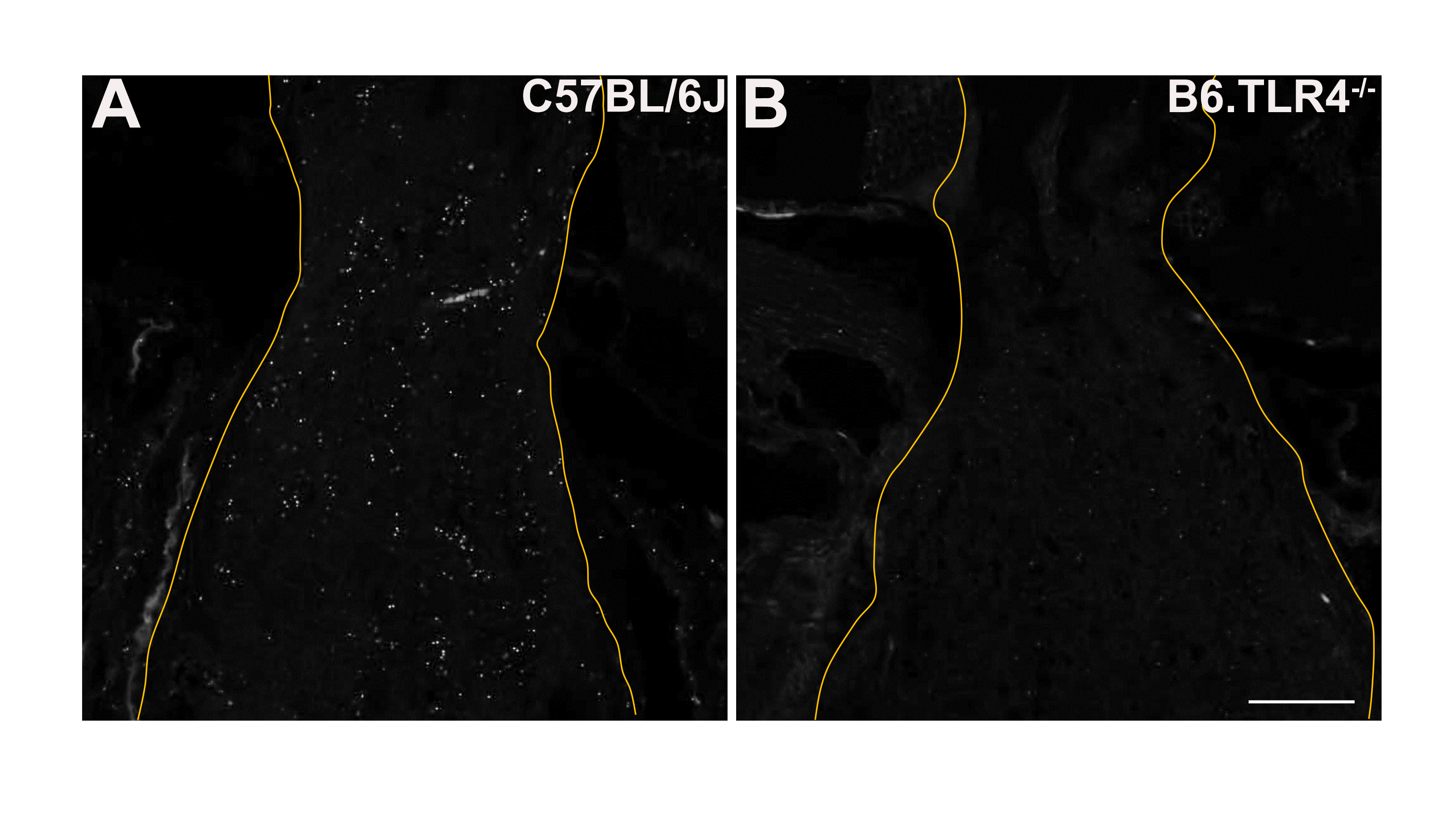

Supplement: Supplementary file 1 — Additional file 1: Figure S1. Mouse Tlr4 mRNA probe is specific. A, B In situ hybridization of Tlr4 in C57BL/6J controls confirms expression of Tlr4 in the mouse ONH, while no specific labeling was detected in Tlr4 knock out (TLR4-/-) mice. [file 13578_2022_800_MOESM1_ESM.tif]
